# Public attitudes towards social media field experiments

**DOI:** 10.1038/s41598-024-76948-z

**Published:** 2024-10-30

**Authors:** Vincent J. Straub, Jason W. Burton, Michael Geers, Philipp Lorenz-Spreen

**Affiliations:** 1 Leverhulme Centre for Demographic Science, Nuffield Department of Population Health, University of Oxford, Oxford, UK; 2 Department of Digitalization, Copenhagen Business School, Frederiksberg, Denmark; 3 Center for Adaptive Rationality, Max Planck Institute for Human Development, Berlin, Germany; 4 Department of Psychology, Humboldt University of Berlin, Berlin, Germany; 5 Public Policy Programme, Alan Turing Institute, London, UK; 6 Faculty of Life Sciences, Humboldt University of Berlin, Berlin, Germany; 7 College of Business, University College Dublin, Dublin, Ireland; 8 Center Synergy of Systems and Center for Scalable Data Analytics and Artificial Intelligence, Dresden University of Technology, Dresden, Germany

**Keywords:** Ethics, Field experiments, Social media, Public attitudes, Survey, Psychology, Human behaviour

## Abstract

The growing use of social media field experiments demands a rethink of current research ethics in computational social science and psychological research. Here, we provide an exploratory empirical account of key user concerns and outline a number of critical discussions that need to take place to protect participants and help researchers to make use of the novel opportunities of digital data collection and field studies. Our primary contention is that we need to elicit public perceptions to devise more up-to-date guidelines for review boards whilst also allowing and encouraging researchers to arrive at more ethical individual study design choices themselves. To ground our discussion in real-world examples of online experiments, we focus on recent social media studies in the field of misinformation, polarization, and hate speech research. We conclude by discussing how we can better strike a balance between meeting ethical guidelines and the concerns of social media users alongside maximizing scientific impact and credibility.

**Supplementary Information:**

The online version contains supplementary material available at 10.1038/s41598-024-76948-z.

## Introduction

Computational social scientists (CSS) studying online human behavior are increasingly conducting field experiments on social media platforms to overcome the limited ecological validity associated with lab experiments^1^. These experiments typically entail administering some treatment to users and analyzing their online behavior before and after the treatment, with or without cooperation of the social media platform^2^. Treatments have involved sending private messages to users^3^, publicly replying to users’ posts^4^, and encouraging users to “like” (subscribe) certain content^5^. In cases where there is platform collaboration, researchers can go further and, for example, alter the algorithmic ranking of content on users’ news feeds^6^. The aim of such studies can range from interventions debunking misinformation, reducing hate speech or boosting people’s ability to spot manipulative techniques^7^ to a real-time assessment of users’ motives for sharing and creating content online^8^.

While social media field experiments have generated exciting results that have appeared in high-impact venues, the innovations they offer also introduce new ethical concerns, such as experimental manipulations in public, networked domains, and sometimes the infeasibility or waiving of informed consent and debriefing for the sake of ecological validity, to name a few. Moreover, their novelty means current ethical guidelines remain underdeveloped for those types of experiments and institutional review boards (IRBs) may be ill-equipped to evaluate associated risks. Not only are these research designs novel, but the social networks themselves are a moving target as their functionality for users can be altered with only small changes^6^. The potential risks of social media studies range from influencing elections^9^ to reducing the willingness to vaccinate^10^. Fortunately, researchers can mitigate some of these risks by adopting more reflexive ethical research practices. For instance, debriefing participants can inform them of the true purpose of the study and help alleviate potential misunderstandings or concerns. Although quantitative estimates are difficult to obtain, ^11^ report that fewer than 30% of papers currently mention any debriefing at all.

Although social media field experiments may typically seem harmless and are likely to be enacted in good faith, the researchers conducting such experiments are currently often left to justify their experimental designs on their own ad hoc, despite potential conflicts with key pillars of research ethics, such as informed consent. Unfortunately, the lack of clear institutional guidance and blurred boundaries for ethical conduct has repeatedly been reflected in public backlash over the past decade. Both older publications, such as the infamous Facebook contagion study^12^, which involved researchers exposing users to fewer positive posts to see if they would lead to greater expressions of sadness, as well as more recent studies, like a large study run on LinkedIn that varied the proportion of weak and strong contacts suggested by its “People You May Know” algorithm^13^, have instigated debate and been met with public criticism. Despite passing an institutional ethical review process or involving private sector scientists collaborating with academic scientists who should have obtained institutional review board approval for all projects they conduct (in the case of the Facebook study), the key point is that both studies were ultimately conducted without users’ awareness. As such, they could have affected some people’s livelihoods and well-being, with potential long-term consequences (e.g., on job prospects), in ways we’ll never fully know, raising questions about transparency and research oversight.

We contend that the public backlash against social media field experiments signals a need to revisit and potentially reform computational social science research ethics, much in the same way that public backlash against biomedical experiments inspired the development of The Belmont Report^14^ in the 1970s^15–17^. Of course, the purpose of research ethics is not to avoid public backlash *per se* and we do not suggest that the research community should be beholden to public opinion alone. However, taking proactive steps to protect (or enhance) the public’s trust in science seem necessary to ensure that important, timely discoveries are well-received when communicated, as demonstrated by the real-world harms that have been induced by anti-science attitudes and lacking trust in vaccine and climate science domains^18,19^. As a first proactive step towards possible research ethics reform, we propose that researchers should engage with the perspective of social media users’ themselves. The incorporation of users’ perspectives could help devise guidelines that effectively protect research participants from harm, especially vulnerable populations (e.g., by shining a light on the actual experiences of participants, rather than experiences inferred by researchers). Moreover, engaging with the public and incorporating the perspectives of affected communities can increase transparency and accountability of research ethics guidelines. Given that it is in the interest of the academic research community to be trusted by the public, openly engaging with and responding to public opinion could serve to foster that trust.

In the present work, we aim to initiate consideration of social media users’ preferences and attitudes towards social media field experiments, focusing on research in domains of misinformation, polarization, and hate speech studies, before considering how these can contribute to the development of revamped guidelines for ethical experiments more broadly. We first unpack the state of affairs by spelling out the problems with existing research practices in more depth. Then we present the results of our survey of public attitudes towards dominant ethical practices, providing a first look at what we can learn when we ask the users’ perspective. Informed by this analysis, which includes public perceptions of high-profile social media field experiments, we then suggest that researchers need to: (1) collect more data on user attitudes towards evolving study designs; (2) better disseminate the values of research impact and scientific reproducibility; and (3) be more transparent about the fact that they are running social media field experiments in the first place. While we focus on academic studies, many of the observations we make also hold true for the online experiments constantly run by private companies (e.g., A/B testing), which often suffer from even less regulatory ethical oversight^20^.

## Pitfalls of current practice

The rapid development in what is technically possible when it comes to designing social media experiments with high ecological validity means new challenging ethical questions are raised continuously. Is it ethical for researchers to place subjects into minimal risk studies without informed consent? What characteristics make an intervention on social media unethical? When does affecting the content users see on social media constitute a form of harm? We are naturally not the first to recognize this or comment on the worrying and persistent lack of institutional attention paid to ethical concerns and public perceptions when it comes to designing online experiments^21^. However, we believe that the potential of current ethical governance procedures to not be fit for meeting the changing nature of research using social media is too urgent an issue to not warrant further investigation, especially from the standpoint of CSS research, a field that can greatly benefit from this new approach to research–if used appropriately.

The reason why current practices appear to not suffice in adequately dealing with social media research, something which has been regularly discussed by research ethicists over the last few years^22–24^, are manifold but ultimately relate to the fact that as technology continues to drive forward, researchers and institutional processes like IRBs can struggle to keep up. As already discussed in a special issue on ethical issues in social media research in 2020^23^, while academic debates about ethical practice seek to catch up with new technological realities and guidelines are suggested by experts and experienced researchers, IRBs and novice researchers can remain unaware of these guidelines and feel uncertain about how to conduct research in this field. This sentiment, of researchers themselves feeling that current procedures are not enough and their demand for additional support, training, and ethics guidance^25,26^ is arguably cause enough to question current procedures.

Given the nascent state of CSS research ethics, prior discussions of how to design ethical social media field experiments have tended to focus on the structural changes that need to take place, such as the establishment of new guidelines, which would benefit the field as a whole. One reoccurring call to action is the suggestion to create new institutions that mirror the state of play in other fields like medical ethics, that is, by creating new committees and codes of practice that can aid researchers in better making the distinction between what constitutes the public and private domain, among other things. Far less attention has, however, been paid to the ethics of particular interventions as they relate to the typical online field experiment. This is likely in part a reflection of the high number of manipulations that researchers can implement when using social media sites, and the difficulty of providing strict ethical guidance for each. To help ground our discussion in real-world examples of online experiments, we focus herein on recent studies in the field of misinformation, polarization, and hate speech research as a case in point^3–5,27^. These usually involve research designs that require users to think about a concept like accuracy and track how an intervention affects real-world sharing of misinformation, or entail survey respondents ranking false content on the likelihood that they believe it to be true and whether would share it^28^.

### Beyond ethics as a box-checking exercise

The first dominant approach to ethical review that arguably epitomizes the current rigid process and stifles progress in researcher engagement is the treatment of ethics as an exercise separate from the research process. Specifically, the tendency of ethics to be viewed by universities and research organizations as something that has to be instituted as an administrative ritual prior to research wherein certain ethics review forms may take precedence over critical judgment, collaboration and the process of ethical inquiry in essence can become what social researchers and critical ethics scholars have previously described as a box-checking exercise^29–32^, given that standard ethics forms required by IRBs consist of batteries of questions, with many being in checklist form. Typically, such forms involve at minimum answering questions addressing: The merit of the project, its scientific rigor, anticipated outcomes, researcher qualifications, consent, reimbursement and risk management considerations, participant recruitment (including vulnerable populations), and data handling. Given these forms are not continuously updated in line with changing methodological possibilities, prescribed standards may quickly become outdated or, as in the context of misinformation, polarization, and hate speech research, forms and guidelines that have been developed primarily for traditional laboratory-style experiments in artificial settings may be blindly adapted to social media field experiments. To be clear, we are not suggesting that researchers do not think about the additional or unprecedented ethical implications of their research at all prior to engagement with IRBs and institutional procedures, nor are we questioning the utility of forms as a way to guide researchers’ critical judgment. Instead, we simply contend that dominant practices of ethical review primarily as an administrative process can stifle engagement if ethics committees do not promote a culture of respectful mutual learning in favor of more rigid top-down processes often epitomized by the use of such forms^33^.

Albeit more speculative, we consider a further danger of standardizing ethical reflection and review as a box-checking exercise to be that it can give the impression that ethical review is a binary process wherein a study that has received IRB approval will not lead to unethical practices or public backlash. Yet, as the exact grounds for why a particular experimental setup was approved are seldom revealed, existing studies may receive ethical approval but could still involve questionable design practices—and no in-depth discussion of why these are justified beyond the claim that they increase ecological validity. While this may sound like a bold claim, it simply follows from the fact that many social science studies with IRB approval recognize that such boards, particularly ones from a different culture to the study participant pool, do not carry out comprehensive ethical assessments.

As discussed in detail by^34^, this concern is underscored by the recognition that standard ethical review processes often overlook the nuanced ethical considerations that arise from the specific context of a study. The ethical standards applied by IRBs may not fully consider cultural or contextual factors that could make certain research practices ethically problematic. The idea that ethical judgments are context-dependent means that practices deemed acceptable in one setting may be inappropriate in another, leading to potential ethical oversights when reviews do not account for these differences. As a result, even research that passes through ethical review may include practices that, while approved, could be ethically contentious or even harmful if the review process fails to adapt to the unique ethical challenges posed by diverse research contexts.

In the context of misinformation, polarization, and hate speech research, it has even been shown experimentally that the entrenched assumption that ethical considerations are met as long as the authors of a study can say that the study has been approved by an IRB may result in interventions (e.g., publicly replying to posts with a fact-check without debriefing users) that increase misinformation sharing and toxicity^35^. This is despite the fact that a careful and thorough debriefing procedure has been shown to help researchers safely and ethically conduct research on these topics^11^.

### Avoiding the rigid commitment to principles

A second approach to ethical review that may enable questionable experimental setups can be argued to result from the fact that many informal recommended best practices are overly concerned with being concept-driven—that is, focused primarily on meeting ethical standards by operationalizing particular principles, such as privacy and autonomy. The roots of this approach can be traced back to the late 1990s and a line of scholarship that sought to refine core concepts relating to research ethics in light of increasing digitization^36^. The issue with this approach (or more accurately, the logic of conceptualization), however, is that it can result in presumptive assumptions, limiting nuanced and contingency-based ethical decision-making. That is, it privileges past practice and historical precedent as the primary guide to ethical reasoning^37^. In other words, by relying first on particular concepts to frame ethics it assumes that these concepts, and historical reasoning, best capture the dilemmas a researcher may face now. Yet, by prioritizing these concepts and assigning them to particular actions or regulations, this approach devalues focusing instead first on the actual and potential consequences of the contemporary actions researchers must take, especially when methods rapidly evolve. An example in the case of misinformation research is taking risk to mean the risk that users would otherwise be exposed to or assuming that consent is given due to the fact that users have agreed to a platform’s terms of service. In reality, we increasingly know that risk on social media is not experienced the same by all users (and researchers) and so depending on the specific intervention, the consequences can vary dramatically^38^. Yet, the heterogeneity of consequences posed by field studies is not accounted for in inflexible concept-driven models of ethical review, which do not encourage researchers to view ethics as a process that can be re-contextualized as needed on a case-by-case basis^37^.

The continued over-reliance on single concepts and little focus on their intersectionality means ethical reasoning can remain a reified process that is resistant or hesitant to modification based upon changes in context, public opinion or technical possibilities. Perhaps the greatest problem with this exclusive reliance on concepts in contrast to contextual details is that it can lead both to misdefining the vulnerability of participants and mistaking predetermined notions with actual experiences of harm^37^. Moreover, it disregards altogether the fact that public opinion can rapidly change when it comes to issues of privacy, vulnerability, anonymity, and harm. Whilst we acknowledge that completely avoiding the potential of harm in online experimentation is difficult (and perhaps impossible), it is perhaps worthwhile remembering that the medical ethics axiom to ‘first do no harm’ has inspired other fields to increase discussions on defining harm and how to recognize it, especially in relation to vulnerable participants, helping to better prepare researchers for when ethical dilemmas do arise^39^.

To take the notion of ‘vulnerability’ as a case in point can help elucidate the above points further. This principle, which is fundamental to ethical research involving human participants, encapsulates the idea that potential risks to subjects must be kept to a minimum, and vulnerable individuals need even greater safeguards, such as pregnant women, incarcerated individuals, and minors. The dominant, concept-driven, categorical use of vulnerability suggests that there is a hierarchy, with certain populations being labeled as vulnerable. As a result, it disregards the degree of vulnerability within these groups, and does not ask researchers to consider and identify the situations in which individuals might be considered vulnerable. Alternative proposals suggest researchers should instead consider this contextual, ‘layered’ intersectional conceptualization of vulnerability^40–42^. Whilst the difficulty of operationalizing this view is often used as a way to justify existing practices, the problems and inconsistencies with the categorical approach are arguably becoming too big to ignore.

In a recent analysis, ^43^ find that, in 355 official documents governing social/behavioral human subjects research across 107 countries, there are 68 distinct vulnerability categories. Their analysis reveals that there are significant differences across different regions and medical classifications are often given too much weight, while important factors like displacement are overlooked. Additionally, there is likely a great deal of diversity within and between these groups. We welcome their and prior suggestions^21^ to focus not only on groups of subjects but the characteristics of research studies and the potential they have to induce or exacerbate vulnerability. Whilst in need-of-testing, ^43^’s TAPIR framework , which outlines the implications of researcher decisions about Topics, steps to Appraise vulnerability, strategies for vulnerability Protections, considerations for Implementation fidelity, and commitments to ongoing Reflection, seems a viable step in the right direction.

## Public attitudes of social media field experiments

Given the issues with the current ethics process, we contend that one sensible step is to more deeply consider the perspectives of those directly affected by social media experiments: The users. For one, users’ attitudes are significant because they can help shape ethical research principles (see^44^ for a notable example regarding public attitudes toward content moderation). Additionally, public acceptance of social media research is crucial for legitimizing and enhancing its effectiveness. Of course, user preferences and attitudes are only one aspect of the broader picture, which must be complemented by established ethical principles to ensure that studies are conducted as ethically as possible. Nevertheless, the research community stands to benefit from understanding what kinds of research practices may be likely to elicit negative public responses, and to understand from which subgroups such responses might come. For example, a researcher designing an experiment with practices that are likely to elicit a negative response could benefit from reflection on that practice and its implementation. If a researcher decides to proceed with a practice that is likely to elicit a negative response, then proactive science communication measures could be taken to, for example, communicate to the public the cost-benefit analyses that have been done throughout the ethical approval process.

To provide more specific guidance to researchers engaged in social media field experimentation, we conducted a pre-registered analysis^1^ of social media users’ perceptions of ethical practices as they relate to the existing high-profile social media studies discussed above. Here we present our findings and provide interpretations of key results.

**Fig. 1:**
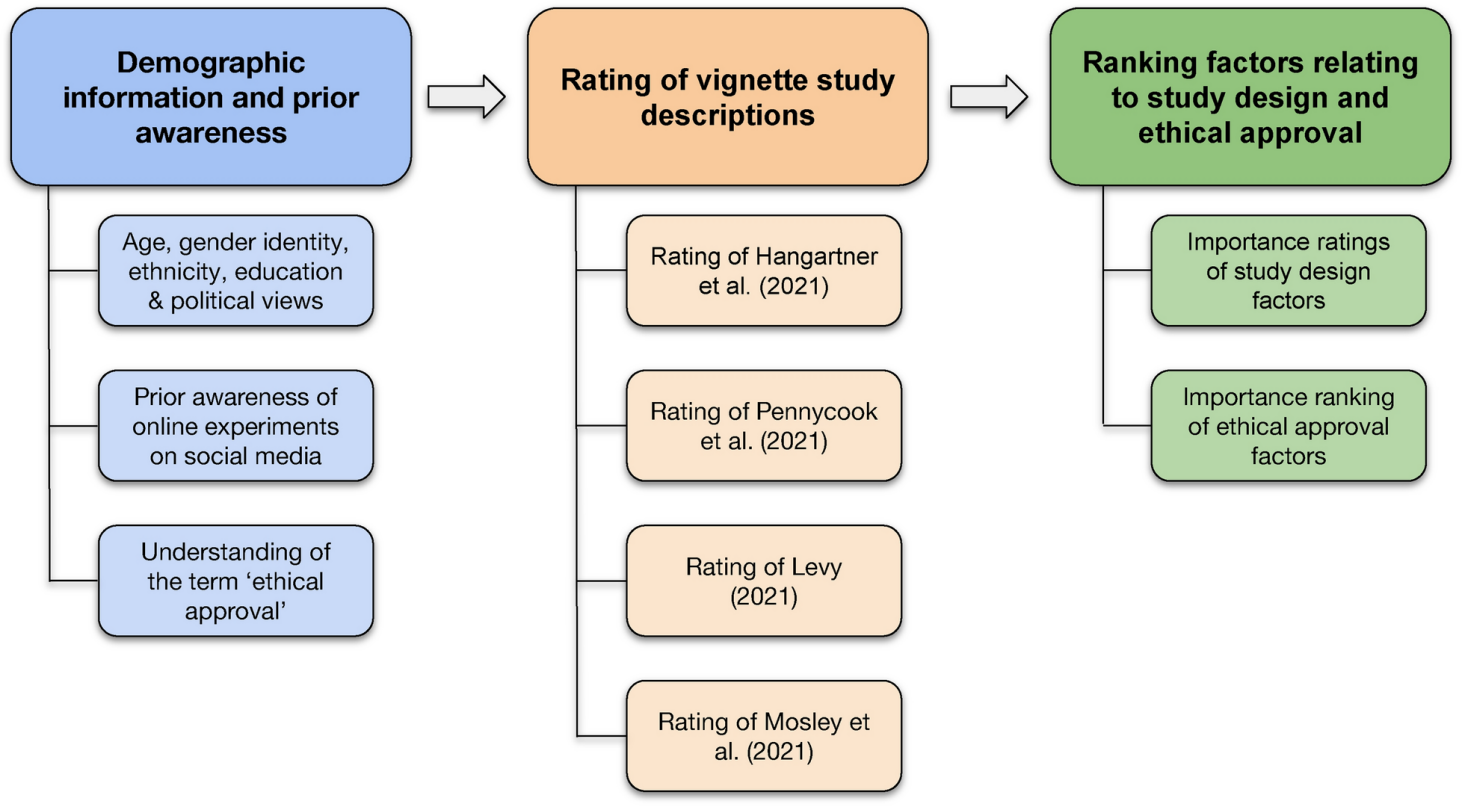
Schematic of study design. The survey questionnaire involved three distinct blocks, including a rating of four vignette study descriptions (in a randomized order) described in the main text. See Supplementary Material Sect. 2 for details.

**Fig. 2:**
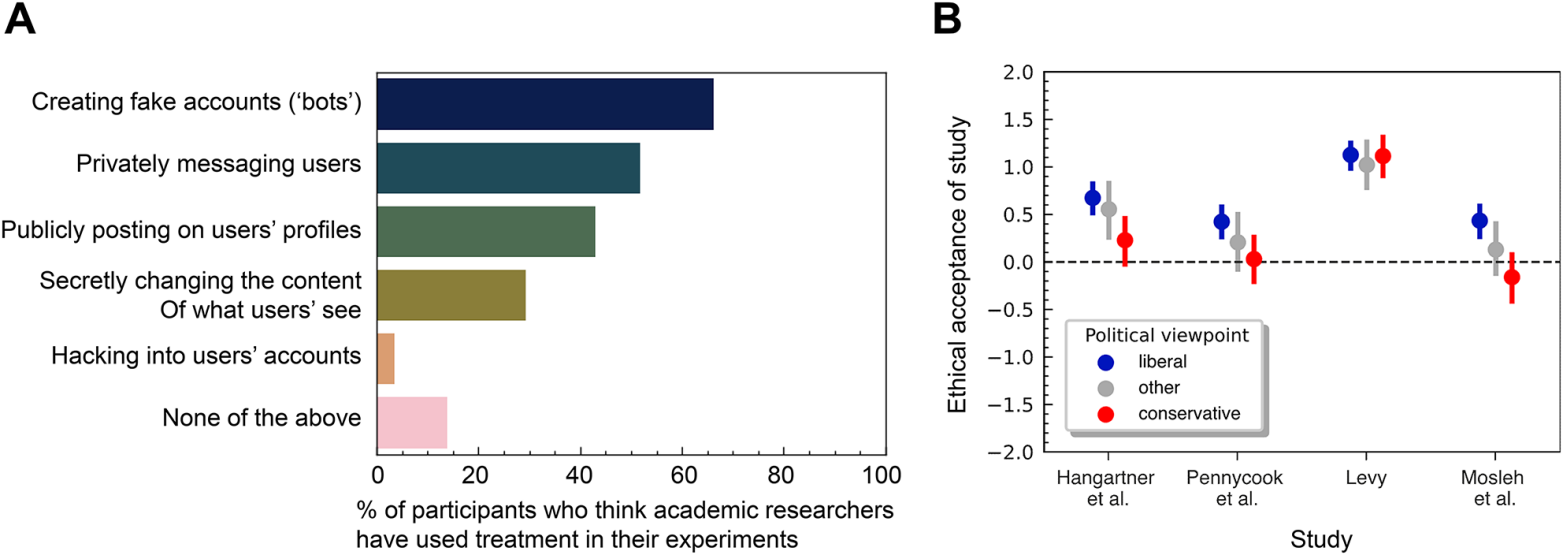
Participant awareness and ratings of vignette study descriptions. (**A**) Participant awareness of treatments administered by researchers. Participants (*N* = 499) were asked “which of the following ways of interacting on social media do you think academics have used in their experiment? (Select all that apply)”. (**B**) Ethical acceptability of four published social media field experiments^3–5,27^. Participants were provided with a brief description of the experiment (approved by the respective authors) and asked “How ethically unacceptable versus acceptable do you find the described study?” using a 5-point rating scale (− 2 = completely unacceptable, 2 = completely acceptable). Points represent means and vertical bars represent standard error. Data is split out by participants’ political viewpoint, such that “liberal” participants ($N=276$) indicated that they are either slightly or very liberal, “conservative” participants ($N=131$) indicated that they are either slightly or very conservative, and “other” participants ($N=92$) indicated that they are neutral or they preferred not to say.

Our study involved running an online survey questionnaire for which we recruited 500 US-based participants aged 18–78 years ($M=41.7$, $SD=13.6$, $41.5\%$ female) via Prolific that were regular users of at least one of Facebook, X (formerly Twitter), or Reddit. Participants (final $N=499$; one participant did not conclude the study) proceeded through three main survey blocks corresponding to three main topics of investigation (Fig. 1). In the first block we explored participants’ awareness of social media field experiments. We find that while users are generally aware that social media data are sometimes used by academic researchers (84.8% were at least “slightly aware”), users are less aware of which interactional treatments researchers have administered. For instance, 66.3% of participants indicated that they were aware that researchers had “created fake accounts (‘bots’)”, 51.8% thought they had conducted experiments involving “privately messaging users”, and 42.9% were aware that researchers had “publicly posted on users’ profiles” (Fig. 2A). Moreover, we find that while users are generally aware that social media data is sometimes used by academic researchers, they are less aware of which interactional treatments researchers have administered. This observation is perhaps unsurprising given the novelty of social media field experiments, yet it underscores the need to consider when specific manipulations should be adopted and how they could be better regulated in part to avoid potential abuses and subsequent reputational damage to the research community.

In the second survey block we asked whether participants perceived actual studies’ procedures to be ethically acceptable—specifically^4^, Study 7 of^3,5^, and^27^^2^. Our findings suggest that participants generally viewed the procedures of past studies to be somewhat ethically acceptable. However, We find that, while participants generally viewed the procedures of past studies to be ethically acceptable, this depended in part on personal user attributes, such as political ideology. For instance, for three of the four studies, users who self-identified as politically conservative tended to view the studies as less positive than those who self-identified as liberal (Fig. 2B). In an unregistered exploratory analysis, we fit a linear mixed-effects model with perceived acceptability (five-point Likert scale responses; − 2: completely unacceptable to 2: completely acceptable) as the dependent variable, political identity (liberal, conservative, or other) as a fixed factor, and random intercepts by participant and stimulus (study). This model revealed that liberals rated the ethical acceptability of experimental designs significantly higher than conservatives ($\beta =0.37$, $SE = 0.10$, $t(496)=3.63$, $p<0.001$), while the difference between neutrals and conservatives was not statistically significant ($\beta =0.21$, $SE=0.13$, $t(496)=1.56$, $p=0.12$). Note, however, that this kind of hypothesis testing was not the primary objective of our study, hence the limited number of stimuli used and imbalanced sample of each political identity (276 participants indicated that they are either slightly or very liberal, 131 indicated that they are either slightly or very conservative, 89 indicated they are either neutral of neither liberal nor conservative, and 3 preferred not to say). This observation could be reflective of anti-academic attitudes being more prevalent among conservatives^45^. However, further hypothesis-driven research seems needed to replicate our observations and distinguish which specific elements of a study design are influencing differences among participants’ acceptability ratings.

Our analysis also serves to illustrate another important consideration often forgotten in more top-down, traditional approaches to ethical review: Participants are seldom a homogeneous group. Being aware of this reminds researchers of the classical ethical principal to serve and take more care of protecting subjects the greater their vulnerability. As highlighted in prior work^46^, this may include paying particular attention to minorities, LGBT individuals, and specific communities; importantly, this also applies to the research process, as awareness and public scholarship present unequal challenges for researchers that echo the differences in user expectations^38^. Our results similarly suggest that if researchers continue running experiments like those considered in our survey, they might contribute to the polarization of liberal versus conservatives attitudes towards science, as conservatives feel more targeted by academic researchers^47^. Yet, to fully understand the effects of various personal user attributes on ethical perceptions of research, more work is clearly needed.

**Fig. 3:**
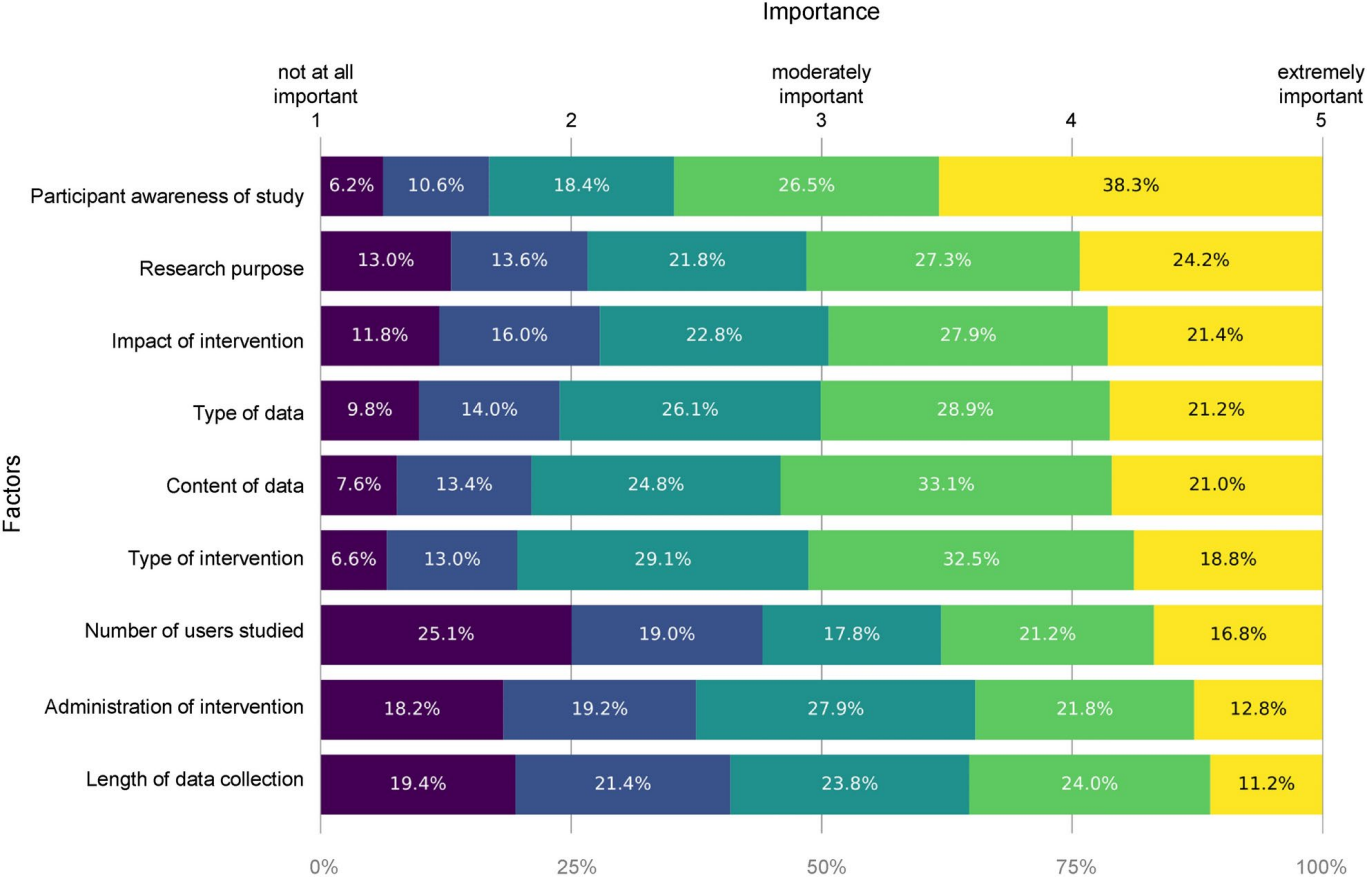
Participant perceptions of the importance of different study design factors. Distribution of participant responses to the question “How important are the following factors for determining your level of concern with academic research conducted on social media, such as online experiments?” using a 5-point rating scale (1 = not at all important, 5 = extremely important). The factors ($n=9$) represent key study design choices identified in prior literature and are depicted in descending order of importance, as measured by the percentage of participants who ranked the factor as ‘extremely important’ (ranging from $38.3\%$ for the top-ranked factor, ‘Participant awareness of study’, to $11.2\%$ for the bottom-ranked factor, ‘Length of data collection’).

An additional observation we make is that participants place a high importance on the type of intervention (treatment) implemented in experiments (i.e., private messaging vs. publicly replying to posts) but care less about its impact (i.e., whether it changed users’ behavior) and how it is administered (e.g., manually by a researcher vs. via an automated account). Moreover, in exploring whether participants judge certain experimental design features to be more important than others in their views of ethical acceptability, we find that users are most concerned about whether study participants have been asked for consent and whether study details have been disclosed. Finally, in the third survey block (importance of study design factors), we explored whether participants judged key experimental design features ($n=9$) identified in prior literature to be more important than others in their views of ethical acceptability. Here, we find that the primary concern is whether participants have been asked for consent and study details have been disclosed to them after completion of the study, with $38.3\%$ of participants ranking ‘Participant awareness of study’ as extremely important, compared to $21.2\%$ for the ‘Type of data’ and $11.2\%$ for the ‘Length of data collection’, for instance (see Fig. 3). Notably, two of the three factors which the smallest percentage of participants ranked as extremely important, ‘Number of users studied’ ($16.08\%$) and ‘Length of data collection’, relate to design features where online and laboratory field experiments differ in practice but not in theory (in contrast to, say, ‘Type of data’ that can be collected, which is a key difference between the two types of design), suggesting that what matters to the public may broadly be the same between the two types of experimental design.

## Discussion: accounting for public perceptions and promoting researcher judgment

Taken together, our results provide empirical support for the need to revamp guidelines for ethically sound social media field experiments. In line with previous surveys^48^, our results affirm that participant awareness is a highly valued ethical principle and—in contrast to earlier commentaries^49^—is considered more important than research purpose and impact. Overall, our findings ultimately lead us to conclude that we need to pay far greater attention to public attitudes than we currently do, especially that users wish to be informed about the use of social media data in scientific studies. While studies on public attitudes towards online field experiments are still sparse, past research and our results further show that these can also be highly contextual, depending on factors such as how the research is conducted or disseminated, who is conducting it, and what the study is about^48^. Research domain, content type, purpose of data use, and awareness of data collection can, for instance, all impact respondents’ comfort^50^. As such, developing new ethical guidelines will require both greater awareness of public perceptions at a population level, and an appreciation of how these may vary across different demographic groups and social media user bases as a result of factors like the specific type of experimental intervention used. Only when IRBs and individual researchers develop a firmer understanding of both of these topics can the CSS community as a whole convincingly claim to be conducting research that is fully in the public interest and strengthen trust in science. This is especially relevant at a time when there is an urgent need to understand the increasing online informational risks users face^51^.

With the above in mind, a final point that we wish to stress is the need for researchers themselves to develop a more informed understanding and judgment of the implications of their study designs. Naturally, all researchers are expected to examine and understand the ethical challenges in their studies’ design, regardless of the field. Yet, new possibilities in social media field experiments mean computational social scientists need to be particularly well-attuned to the changing benefits and risks of experimenting on platforms like X (formerly Twitter), Facebook or Reddit. For example, ongoing work on (mis)information sharing goes beyond the popular approach to link users’ survey responses (e.g., demographics or political views) with their digital footprints^52^, but additionally elicits users’ behavioral motives “in the moment”^53^. Given the dynamic nature of social media, a developed sense of critical judgment on the part of researchers is arguably becoming only more important. Relatedly, given the changing relationship the public holds with regards to social media, the other important ingredient that is arguably missing from the current landscape of research ethics is a greater ongoing researcher awareness of public attitudes to current practices and specific interventions.

Although there is a growing recognition that the relationship between IRBs, researchers, and the public should be one of continuing cooperative dialogue^37^, we contend that this combination of (1) more informed researcher judgment, and (2) more engagement with public sentiment is what is needed to ensure future experiments are more ethically sound and publicly acceptable. To be clear, we are not suggesting that current practices (i.e., the tendency to leave ethical review to IRBs) are dropped wholesale; rather, we wish to stress how they can be augmented through the adoption of these additional practices. In practice, this means researchers engaging in deeper, bottom-up ethical reflection when it comes to the day-to-day practices of designing a study, regardless of the discipline, country and context they work in^37^. Ethical reflection is here taken to entail spending more time personally grappling with specific questions related to the experiment setup at hand, before and after study completion, such as: To what extent can the current intervention be justified? How may it exacerbate the vulnerability of participants? Are there alternative methodologies that have not been considered? As described by^43^ and their TAPIR framework, researchers can do so by considering how the topic of study might introduce new vulnerabilities, appraise how an intervention affects individuals in different country contexts, devise and implement relevant protections to account for any new vulnerabilities, and reflect on or ideally verify that no new vulnerabilities were introduced to appropriate steps were taken to address them.

In spending more time wrestling with questions about the ethical nature of their studies, alongside consulting IRB guidelines, researchers should be encouraged to embrace ethical pluralism and cross-cultural awareness, allowing them to iterate over design choices while accepting the possibility of multiple, ethically legitimate judgment calls—similar to public attitudes towards field experiments. Importantly, this call for further training in research ethics is not meant as a way to prevent the design of future studies like the ones we use in our survey here, regardless of how ethical they are. Rather, it is a low-cost and easy to implement practical preventative measure that arguably not only has inherent value as a pedagogical practice but ultimately benefits research in the long run, by fostering collaboration and ensuring researchers are aware of and conduct research on changing social and ethical concerns, among other factors^54^. Moreover, given the potential of large language models to transform CSS^55^, including in experimentation, and usher in further ethical quandaries, training in research ethics can perhaps also be seen as a necessary preventative measure to foster greater awareness of the potential perils of AI-assisted experimentation in the next generation of CSS students; something that is already regularly discussed in the field of computer science and cybersecurity^56^ and has been readily observed in the integration of AI in social media^57^.

## Concluding thoughts

Taken together, our discussion and the results of our survey lead us to emphasize that a number of critical discussions and decisions still need to happen to ensure researchers conducting social media field experiments do so in a way that is ethically sound and publicly acceptable. Whilst Kinder-Kurlanda and Zimmer conclude that, “there is no ‘ethical research’ of the internet in the social age ... we can really only strive for ‘ethically-informed’ research practices”^58^, we believe there is a way forward. A first step in the right direction is further data collection on user attitudes, both to get a more comprehensive picture of differences amongst different subpopulations and to paint a clearer landscape of the public acceptability of novel interventions. Secondly, the research community as a whole arguably needs to better disseminate the values of research impact and scientific reproducibility, as achieving greater ecological validity will naturally mean that studies are more meaningful—allowing any findings that can be translated to policies to ultimately benefit the public more in the long term. Third, research organizations and researchers arguably need to begin being more proactively transparent about the fact that they are running social media field experiments, as a sizable proportion of the public appears to remain unaware of this and some appear to consistently prefer to be excluded from field experiments altogether.

Importantly, the considerations and recommendations that we discuss here are not limited to those conducting misinformation, polarization, and hate speech research. Our primary objective is to guide all researchers wishing to conduct field experiments on social media in choosing a research design that is the result of considered ethical reflection, abides by existing ethical review standards, and is in tune with public sentiment. In the long term, ensuring studies are aligned with public sentiment will ensure trust and funding in CSS is maintained, benefiting both the public and researchers. Thus, explicitly eliciting public perceptions of study designs will both help protect participants and aid researchers in maximizing the scientific impact and credibility of their work.

## Supplementary Information

Supplementary Information.

## Data Availability

This study and all experimental protocols were reviewed and approved by the Max Planck Institute for Human Development in accordance with the procedures laid down by the Max Planck Society for ethical approval of all research involving human participants. Informed consent was obtained from all subjects (see Supplementary Material Sect. 1 for details) and all methods were carried out in accordance with relevant guidelines and regulations. Our main research questions, survey design, and analyses were pre-registered before the collection of the data (AsPredicted: #103940). The datasets generated and analyzed during the current study are available in a Open Science Framework repository: https://osf.io/swcvq/. Data and code are also available on GitHub at the following URL: https://github.com/vincejstraub/article-digex-survey. Participants were recruited using the Prolific platform. The survey responses were recorded using Qualtrics software and analyzed in the R statistical language and Python.

## Footnotes

1 The pre-registration for our study is available on the Open Science Framework at: https://osf.io/swcvq/.

2 These studies were selected due to their publication in high-profile venues and because their experimental designs involve a range of different treatments (e.g., private messages, public replies, browser extensions, use of “bot” accounts, etc.). The standardized-vignette study descriptions presented to participants in our survey were approved by the respective authors for accuracy (see Supplementary Material Sect. 2 for details).
