# Author Correction: Public attitudes towards social media field experiments

**DOI:** 10.1038/s41598-025-89155-1

**Published:** 2025-02-12

**Authors:** Vincent J. Straub, Jason W. Burton, Michael Geers, Philipp Lorenz-Spreen

**Affiliations:** 1 Leverhulme Centre for Demographic Science, Nuffield Department of Population Health, University of Oxford, Oxford, UK; 2 Department of Digitalization, Copenhagen Business School, Frederiksberg, Denmark; 3 Center for Adaptive Rationality, Max Planck Institute for Human Development, Berlin, Germany; 4 Department of Psychology, Humboldt University of Berlin, Berlin, Germany; 5 Public Policy Programme, Alan Turing Institute, London, UK; 6 Faculty of Life Sciences, Humboldt University of Berlin, Berlin, Germany; 7 College of Business, University College Dublin, Dublin, Ireland; 8 Center Synergy of Systems and Center for Scalable Data Analytics and Artificial Intelligence, Dresden University of Technology, Dresden, Germany

Correction to: *Scientific Reports* 10.1038/s41598-024-76948-z, published online 30 October 2024

The original version of this Article contained an error in Figure 2, where the bars in panel A were interchanged.

The original Figure 2 and accompanying legend appear below.

**Fig. 2:**
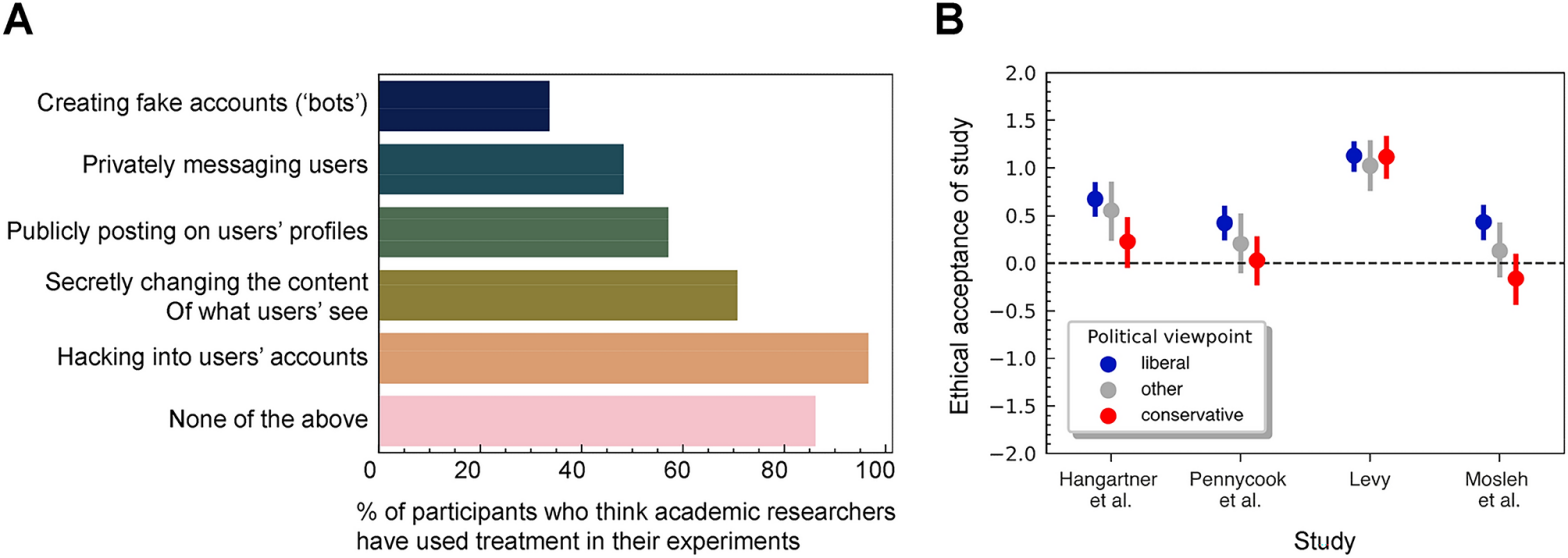
Participant awareness and ratings of vignette study descriptions. (**A**) Participant awareness of treatments administered by researchers. Participants (*N* = 499) were asked “which of the following ways of interacting on social media do you think academics have used in their experiment? (Select all that apply)”. (**B**) Ethical acceptability of four published social media field experiments^3,4,5,27^. Participants were provided with a brief description of the experiment (approved by the respective authors) and asked “How ethically unacceptable versus acceptable do you find the described study?” using a 5-point rating scale (− 2 = completely unacceptable, 2 = completely acceptable). Points represent means and vertical bars represent standard error. Data is split out by participants’ political viewpoint, such that “liberal” participants $N=276$ indicated that they are either slightly or very liberal, “conservative” participants $N=131$ indicated that they are either slightly or very conservative, and “other” participants $N=92$ indicated that they are neutral or they preferred not to say.

The original Article has been corrected.

