# Supplementary material for: Public attitudes towards social media field experiments
Source: Sci Rep. 2024 Oct 30;14:26110. doi: 10.1038/s41598-024-76948-z (PMC11525638; doi:10.1038/s41598-024-76948-z)
Supplement: Supplementary file 1 — Supplementary Information. [file 41598_2024_76948_MOESM1_ESM.docx]

Supplementary Material for: Towards more ethical social media field experiments

Vincent J. Straub^1,5,^, Jason Burton^2,3^, Michael Geers^3,4^, and Phillip Lorenz-Spreen^3^

^1^ Public Policy Programme, Alan Turing Institute, London, United Kingdom

^2^ Department of Digitalization, Copenhagen Business School, Frederiksberg, Denmark

^3^ Center for Adaptive Rationality, Max Planck Institute for Human Development, Berlin, Germany

^4^ Department of Psychology, Humboldt University of Berlin, Berlin, Germany

^5^ Leverhulme Centre for Demographic Science, Nuffield Department of Population Health, University

of Oxford, Oxford, United Kingdom

To whom correspondence should be addressed: [vincent.straub@ndph.ox.ac.uk](mailto:vincent.straub@ndph.ox.ac.uk)

# Contents

1. [**Study information and informed consent form**](#_bookmark0) **2**
2. [**Survey questionnaire**](#_bookmark1) **3**

# 1 Study information and informed consent form


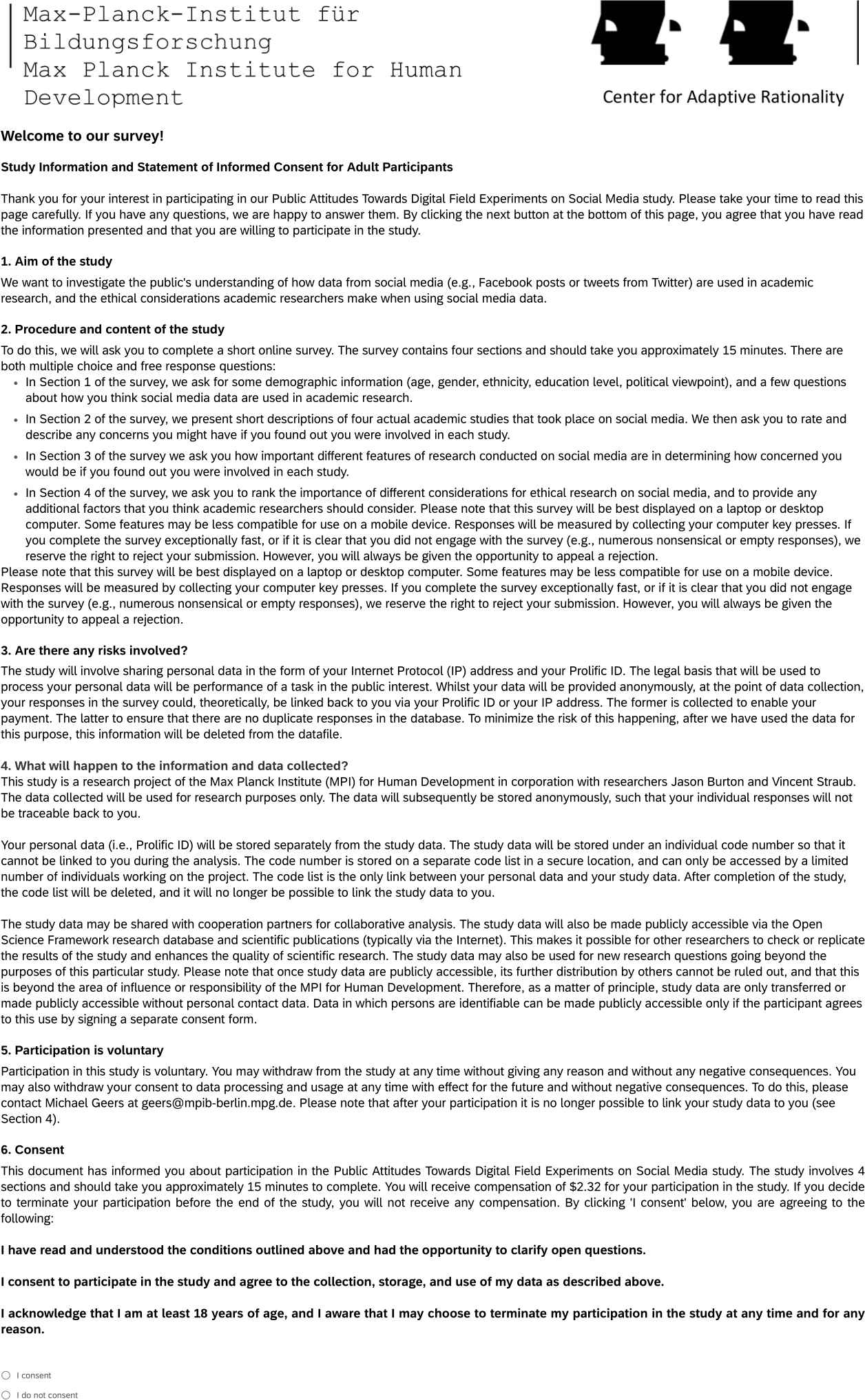


Figure 1: **Screenshot of the study information and informed consent form.**

# 2 Survey questionnaire

**Section 1: Demographics and prior awareness**

1. **How old are you?**:
2. **How do you describe yourself?**

◯ Female

◯ Male

◯ Non-binary/third-gender

◯ Prefer to self-describe:

◯ Prefer not to say

1. **What is your ethnic background?**

◯ African-American

◯ Asian – Eastern

◯ Asian – Indian

◯ Hispanic

◯ Mixed race

◯ Native American

◯ White/Caucasian

◯ Other:

◯ Prefer not to say

1. **What is the highest level of education you have achieved?**

◯ Highschool

◯ Bachelor’s degree

◯ Master’s degree or above

◯ Other:

◯ Prefer not to say

1. **What is your political viewpoint?**

◯ very conservative

◯ slightly conservative

◯ Neutral/Neither conservative or liberal

◯ Slightly liberal

◯ Very liberal

◯ Prefer not to say

1. **Are you aware that academic researchers sometimes use social media data (e.g. tweets from Twitter) for research purposes?**

◯ Not at all aware

◯ Slightly aware

◯ Moderately aware

◯ Very aware

◯ Extremely aware

1. **Which of the following do you think are advantages of social media data that account for why academics collect them for their research? (Select all that apply)**

**Social media data...**

◯ ... are large and can obtain millions of data points

◯ ... reflect events in real-time and can be collected continuously over time

◯ ... are naturalistic in that they do not require researchers to directly interact with research volunteers

◯ ... often capture social relationships not found using traditional methods (e.g. surveys)

◯ ... are readily accessible to researchers and easy to collect

◯ ... are always representative of people’s offline behavior

◯ ... are unaffected by the way social media platforms work

◯ ... are well formatted and never contain any missing data

◯ None of the above

1. **Sometimes academic researchers run online experiments on social media sites that in- volve interacting with users. Which of the following ways of interacting on social media do you think academics have used in their experiments? (Select all that apply)**

◯ Privately messaging users

◯ Publicly posting on users’ profiles

◯ Creating fake accounts (“bots”)

◯ Secretly changing the content of what users see

◯ Hacking into users’ accounts

◯ None of the above

1. **Which of the following research topics do you think academic researchers have used social media data for? (Select all that apply)**

◯ Political elections (e.g. voting behavior)

◯ Economic forecasting

◯ Presidential approval ratings

◯ Health topics (e.g. spread of diseases)

◯ Well-being and economic satisfaction

◯ Communication (e.g. spread of opinions and hate speech)

◯ Public sentiment (e.g. environment-related concerns)

◯ News consumption (e.g. sharing of misinformation)

◯ Social networks

◯ None of the above

1. **In order to run their experiments, academic researchers often need to get ethical ap- proval from their institution. Please briefly describe what you think ‘ethical approval’ means?**

# Section 2: Study descriptions

You have completed Section 1 of the survey.

In Section 2 of the survey you will be presented with short descriptions of actual academic stud- ies that took place on social media. You will then be asked to rate and describe any concerns you might have if you found out you were involved in each study.

Each of the described studies either received ethical approval from the researchers’ institution, or was deemed to be exempt from the ethical review process.

**Study 1 description**

*A team of academic researchers wanted to see if they could reduce hate speech on social media, so they conducted an experiment on Twitter. First, the researchers identified over one thousand thousand Twitter users who had previously made public posts containing hate speech. Then the researchers created human-operated anonymous Twitter accounts and used these to pub- licly reply to users’ posts containing hate speech with different kinds of messages. Finally, the researchers analyzed the users’ 1,000 public posts before receiving the public reply, and the users’ public Twitter behavior for 4 weeks after receiving the public reply. Participants were not aware they were part of a research study. The researchers found that users who received a public reply emphasizing empathy posted less hate speech and were more likely to delete their hate speech post that received the reply.*.

1. **How ethically unacceptable versus acceptable do you find the described study?**

| Completely | Somewhat |  | Somewhat | Completely |
| --- | --- | --- | --- | --- |
| unacceptable | unacceptable | Neutral | acceptable | acceptable |

◯ *− − − − − − −* ◯ *− − − − − − −* ◯ *− − − − − − −* ◯ *− − − − − − −* ◯

1. **Please describe any thoughts or concerns you have about the study.**
2. **Is there any additional information about the study or researchers that would influence how ethically acceptable you find it?**

**Study 2 description**

*A team of academic researchers wanted to see if they could reduce the sharing of misinforma- tion on social media, so they conducted an experiment on Twitter. First, the researchers identi- fied over five thousand Twitter users who had previously made public posts containing links to right-leaning (i.e., politically conservative) websites that professional fact-checkers had rated as untrustworthy. Then the researchers created human-operated anonymous Twitter accounts to send each of the identified users an unsolicited private message asking them to rate the ac- curacy of a single non-political headline. Finally, the researchers analyzed all of the posts the users shared publicly on Twitter within 24 hours of receiving the private message. Participants were not aware they were part of a research study. The researchers found that users shared higher-quality news content after being sent the private message.*

1. **How ethically unacceptable versus acceptable do you find the described study?**

| Completely | Somewhat |  | Somewhat | Completely |
| --- | --- | --- | --- | --- |
| unacceptable | unacceptable | Neutral | acceptable | acceptable |

◯ *− − − − − − −* ◯ *− − − − − − −* ◯ *− − − − − − −* ◯ *− − − − − − −* ◯

1. **Please describe any thoughts or concerns you have about the study.**
2. **Is there any additional information about the study or researchers that would influence how ethically acceptable you find it?**

**Study 3 description**

*An academic researcher wanted to see if the type of content people view on social media influ- ences political polarization, so they conducted an experiment on Facebook. First, the researcher used a targeted advertisement on Facebook. Over thirty thousand Facebook users decided to take part in the study. Participants were informed that they would be part of a research study. At the end of the survey, each user was encouraged to ”Like” the Facebook pages of either liberal news outlets (e.g., MSNBC) or conservative news outlets (e.g., Fox News). Eight thousand users who completed the survey were also offered to install a web browser extension, which allowed the researcher to record the news websites users visited with that browser and all the posts the users viewed in their Facebook news feeds for up to eight weeks. For installing the extension, users could choose between receiving a $5 gift card, participating in a lottery for a $200 gift card, or receiving a copy of the study results. The researcher found that users who subscribed to the Facebook pages of news outlets that opposed their initial political views subsequently visited those outlets more and reported less negative attitudes towards the opposing political party, but did not change their political views.*

1. **How ethically unacceptable versus acceptable do you find the described study?**

| Completely | Somewhat |  | Somewhat | Completely |
| --- | --- | --- | --- | --- |
| unacceptable | unacceptable | Neutral | acceptable | acceptable |

◯ *− − − − − − −* ◯ *− − − − − − −* ◯ *− − − − − − −* ◯ *− − − − − − −* ◯

1. **Please describe any thoughts or concerns you have about the study.**
2. **Is there any additional information about the study or researchers that would influence how ethically acceptable you find it?**

**Study 4 description**

*A team of academic researchers wanted to see if they could reduce the sharing of misinfor- mation on social media, so they conducted an experiment on Twitter. First, the researchers identified two thousand Twitter users who had made posts containing links to news articles that professional fact-checkers had marked as containing false claims. Then the researchers created automated, human-looking accounts that automatically publicly replied to the posts of users by stating that the tweet might not be true and providing a link to a fact-checking website. Finally, the researchers analyzed all the content the users shared publicly on Twitter via tweets in the two weeks before and in the 24 hours after receiving the public reply. Participants were not aware they were part of a research study. The researchers found that the public reply decreased the quality, and increased the partisan slant and language toxicity, of users’ subsequent retweets.*

1. **How ethically unacceptable versus acceptable do you find the described study?**

| Completely | Somewhat |  | Somewhat | Completely |
| --- | --- | --- | --- | --- |
| unacceptable | unacceptable | Neutral | acceptable | acceptable |

◯ *− − − − − − −* ◯ *− − − − − − −* ◯ *− − − − − − −* ◯ *− − − − − − −* ◯

1. **Please describe any thoughts or concerns you have about the study.**
2. **Is there any additional information about the study or researchers that would influence how ethically acceptable you find it?**

You have completed Section 2 of the survey. Please proceed to Section 3.

# Section 3: Ranking study design features

1. **How important are the following factors for determining your level of concern with academic research conducted on social media, such as online experiments?**

**Content of data**

(i.e., i.e., whether the collected data contains the posts made by users or the content they see on their newsfeed etc.)

| ◯ | ◯ | ◯ | ◯ | ◯ |
| --- | --- | --- | --- | --- |
| ◯ | ◯ | ◯ | ◯ | ◯ |

**Number of users studied**

(i.e., whether the social media posts of one or five thou- sand users are collected)

**Research purpose**

(e.g., whether the focus of the study is to reduce hate

Not at all important

- - - Extremely

important

speech online or understand how social media influences people’s political opinions)

**Type of data**

(i.e., whether the collected data are the links of websites or individual Twitter posts etc.)

**Length of data collection**

(i.e., is users social media behavior monitored for days or weeks?)

**Administration of intervention**

(e.g., whether the intervention is carried out by manually by a researcher or automatically by an automated Twitter account)

**Type of intervention**

(i.e., does the research involve privately messaging users, publicly replying to posts, or asking users to subscribe cer- tain pages, etc.?)

**Participant awareness of study**

(i.e., does the researcher gain consent and disclose study details after the study is complete?)

**Impact of the intervention**

(i.e., did the intervention change the social media behavior of users?)

◯ ◯ ◯ ◯ ◯

| ◯ | ◯ | ◯ | ◯ | ◯ |
| --- | --- | --- | --- | --- |
| ◯ | ◯ | ◯ | ◯ | ◯ |
| ◯ | ◯ | ◯ | ◯ | ◯ |
| ◯ | ◯ | ◯ | ◯ | ◯ |
| ◯ | ◯ | ◯ | ◯ | ◯ |
| ◯ | ◯ | ◯ | ◯ | ◯ |

1. **Are there any other aspects of research conducted on social media that are important for determining your level of concern?**
2. **To run experiments like the ones described in the previous section of this survey, aca- demic researchers often need to get ‘ethical approval’ from a board of reviewers at their institution. This requires the researchers to explain and justify things like how they will handle the data they collect, how they will protect the anonymity of partic- ipants, and how they will limit any risks imposed on participants before starting an experiment.**

**In your opinion, which of the following factors should be the most important for re- searchers to address in order to receive ethical approval?** Click and drag the factors into your desired order from 1 (most preferred) to 7 (least preferred).

- 1. **Scientific reproducibility** – the experiment design should be easy for other researchers to reproduce; for example, to check that the results are robust and can be trusted
  2. **Respect for participants via informed consent** – all participants involved in the ex- periment must have been given information about the experiment and made an explicit decision to be involved in the study, and have the right to withdraw from the experiment at any time
  3. **Just and fair study design** – one group of participants should not bear the costs of re- search while another group reaps its benefits, study participants should also be appropri- ately compensated
  4. **Safeguard participant anonymity** – individual participants must not be identifiable; data cannot be traced back to individuals
  5. **Avoid harms at all costs** – researchers should not conduct studies which carry the risk that a participant will be harmed regardless of the benefits that might come to others
  6. **Balance benefits versus risks** – researchers should try to reduce the risk profile of a study by ensuring the benefits of the study are maximized and the risks are minimized
  7. **Respect the law and wider public Interest** – researchers should attempt to identify and obey relevant laws, contracts, and terms of service and should be clear about their goals, methods, and results at all stages of their research, taking responsibility for their actions

1. **Are there any additional factors that you think should be considered for researchers to receive ‘ethical approval’ from their institutions before running experiments on social media? If so, please add them to the table below by providing a brief description and mentioning where they would be placed when added to your ranking of the 7 factors above from 1 (most preferred) to 10 (least preferred), before clicking next.**

Position in ranking (1-9)

Description of additional factor

[ ]

Description of additional factor

[ ]

Description of additional factor

[ ]

We thank you for your time spent taking this survey. Your response has been recorded.
